# Differential cold stress intensities drive unique morphological and transcriptomic changes in *Zea mays* root hairs

**DOI:** 10.1186/s12864-025-12001-1

**Published:** 2025-09-22

**Authors:** Mauritz Leonard Sommer, Yaping Zhou, Frank Hochholdinger

**Affiliations:** Institute for Crop Science and Resource Conservation, Crop Functional Genomics, University of Bonn, Bonn, 53113 Germany

**Keywords:** Abiotic stress, Cold, Maize, RNA-seq, Root hair, Transcriptome

## Abstract

**Background:**

Maize seedlings grown in temperate climates often encounter suboptimal temperatures that delay seedling establishment and reduce crop productivity. While research commonly treats cold stress as a uniform phenomenon, the nuances between moderate chilling and more extreme cold have rarely been disentangled. Dissecting these distinct temperature thresholds will uncover the adaptive strategies essential for maize resilience under varying low-temperature conditions.

**Results:**

We investigated how mild (16 °C/12 °C) and severe (10 °C/6 °C) cold stress intensities modulate maize root hair development by integrating phenotypic observations with transcriptome profiling. Mild cold imposed partial inhibition on both primary root and hair growth. Mild cold stress induced fewer differentially expressed genes, mostly downregulated, affecting multiple processes including cell wall remodeling, while severe cold stress completely inhibited hair elongation and triggered a broader transcriptional reprogramming, highlighted by strong induction of peroxidases and pronounced changes in hormone signaling cascades (auxin, jasmonate, ethylene, cytokinin). While mild cold still maintained limited root hair elongation, severe cold repressed key root hair developmental regulators (e.g., *rhd6*, *rsl4*) and auxin transporters.

**Conclusion:**

This study demonstrated that maize root hairs respond to cold stress in a severity-dependent manner. Mild cold triggers adaptive remodeling that preserves limited growth, while severe cold prioritizes stress defense, culminating in more pronounced morphological inhibition. By defining the transcriptomic and morphological changes linked to each cold intensity, this study provides novel insights that could be applied for breeding strategies aimed at strengthening early seedling vigor and cold resilience in maize.

**Supplementary Information:**

The online version contains supplementary material available at 10.1186/s12864-025-12001-1.

## Background

Domesticated in Central America about 10,000 years ago [1], maize became a mainstay of global agriculture, providing a fundamental resource for food, feed and industrial applications [2]. With cultivation spanning from tropical to temperate regions, maize shows the broadest geographic distribution among major crops [3, 4]. Early planting in maize cultivation can extend the growing season, foster vigorous vegetative growth and enable earlier harvests, which collectively minimizes yield losses from summer droughts in key developmental stages [5, 6]. However, the temperate climates of Central and Northern Europe present unique challenges, particularly due to the prevalence of cold stress during the critical early growth phases. Cold stress, defined by temperatures that fall below the optimal growth range of 21 °C to 27 °C, affects maize through delayed seedling emergence, reduced germination rates and compromised plant vigor, ultimately leading to a decrease in yield [7, 8]. The advent of climate change, characterized by increased frequency of extreme weather events, exacerbates these challenges, underscoring the urgent need for robust cold tolerance in maize [9].

Cold temperatures reduce the hydraulic conductance of roots [10, 11], which negatively impacts water relations and nutrient uptake, thereby restricting the growth potential of plants [12]. In response, plant cells increase the production of compatible solutes such as carbohydrates and specific amino acids, crucial for maintaining cellular water potential and protecting against dehydration [13]. Moreover, low temperature inhibits the growth of the primary root and reduces the branching angles between primary and lateral roots [14, 15]. In maize, epidermis cells are the cell type most sensitive to cold with the highest number of differentially expressed genes after cold exposure [16]. Root hairs are tubular outgrowths of epidermal cells which increase the surface area of the root, enhance nutrient and water uptake and support plant anchorage [17, 18]. Thus, root hairs are instrumental for the adaptation and resilience of roots under cold conditions.

The molecular and physiological responses of maize to cold stress involve a dynamic interplay of metabolic and gene expression changes aimed at maintaining homeostasis and growth under suboptimal conditions [8, 19]. Cold stress significantly affects gene expression, leading to the activation of pathways associated with transcription, stress signaling and cell-wall organization [20]. Transcriptional changes regulated by the INDUCER OF CBF EXPRESSION (ICE) signaling cascade are a central mechanism orchestrating a comprehensive cold response. Key transcription factors, such as C-REPEAT BINDING FACTOR/DRE BINDING FACTOR1 (CBF/DREB1) and DREB2, play a pivotal role in the response and tolerance of plants to cold stress by regulating cold-regulated (COR) genes [21]. Furthermore, DREB2.1 negatively regulates root hair plasticity at low temperatures by coordinating the expression of genes related to root hair elongation [16]. Phytohormones interact with the ICE-CBF-COR pathway, acting both as regulators and targets within this network, to modulate plant responses to cold stress [22]. In particular, salicylic acid, ethylene and abscisic acid play crucial roles in plant stress response [23].

Reactive oxygen species (ROS) are highly reactive and can damage lipids, proteins, carbohydrates and DNA, thus compromising cell viability [24]. Their accumulation is a common response to various abiotic stresses, including cold stress. Plants produce most ROS in chloroplasts, mitochondria and peroxisomes. Under cold conditions, both photosynthetic electron transport in leaves [25] and the mitochondrial electron transport chain [26] are particularly susceptible to overreduction, thereby exacerbating ROS generation. Cell walls and membranes serve as secondary sites of ROS accumulation [27]. At optimal levels, ROS act as key signaling molecules, driving cell wall remodeling and promoting root hair elongation. However, excessive ROS accumulation under stress conditions inhibits root hair growth by causing oxidative damage [28]. Chilling activates various defense mechanisms. Among those, modification of membrane composition and cell-wall organization aims at stabilizing cellular structures [20, 29]. This process often involves alterations of the lipid composition to enhance membrane fluidity [30] and the accumulation of dehydrins. Dehydrins are hydrophilic proteins known for their protective effects on cellular membranes [31]. Cold stress also triggers pectin remodeling, increasing methylesterification to strengthen the cell wall, while adjustments in hemicellulose and xyloglucan enhance flexibility. Lignin deposition further reinforces the wall, reducing the risk of structural damage during freezing [27, 32]. Finally, plants produce cold shock proteins, which facilitate RNA processing and enhance translation efficiency at low temperatures [33].

In the suboptimal temperature range for maize growth (20 °C and below), the impact of cold stress on maize can be categorized as moderate chilling stress (10−20 °C), characterized by growth reduction and severe chilling stress (0−10 °C), defined by cellular and tissue injury [34]. However, the actual severity of cold stress in maize can vary depending on the cold acclimation status [35] and the inherent cold tolerance of the cultivar [36]. Root growth in maize recovers from severe cold by the expansion of cells newly produced by the meristem [37]. Extension growth ceases at the level of each individual expanding cell once it experiences severe cold. Growth recovery from severe cold does not reverse this effect but requires the generation of fresh cells [37].

We hypothesized that cold stress triggers distinct transcriptomic shifts in maize root hair cells, driving morphological adaptations that mitigate growth constraints under suboptimal temperatures. To test this, we compared mild and severe cold stress in the maize inbred line B73, focusing on gene expression and phenotypic alterations in root hairs. Specifically, our objectives were to: (1) characterize the molecular pathways affected by varying cold intensities and (2) determine how these pathways influence root hair development. This integrated phenotypic and transcriptomic approach provides insights into the molecular basis of cold tolerance.

## Methods

### Plant material, growth conditions and phenotypic measurements

We cultured seedlings of the maize inbred line B73 in germination paper rolls (Anchor Paper Co., Saint Paul, USA), following methodologies outlined in prior studies [38, 39]. We placed the paper rolls vertically in 5 L buckets, each containing approximately 1.5 L of distilled water, and covered the buckets with aluminum foil on their sides and bases to reduce the exposure of the roots to diffused light [40]. The seedlings were grown in growth cabinets (Conviron, Winnipeg, Manitoba, Canada) under control conditions [41] (27 °C/22 °C; 16 h light/8 h dark) for 3 days before applying any further treatments. Subsequently, we subjected the seedlings to different temperature treatments for 24 h. A 16 h light/8 h dark cycle including a temperature reduction to simulate night conditions was selected to mimic the naturally fluctuating soil temperatures during early establishment of seedlings [15]. The selected mild cold treatment temperature of 16 °C/12 °C aimed to mitigate severe root tissue damage typically observed below 10 °C [34], while still triggering physiological responses to cold stress. The severe cold treatment of 10 °C/6 °C aimed to elicit a stronger response from the seedlings without being phytocidal.

High resolution photographs of all seedlings were taken after the 3-day germination period and after each treatment using a Sony a7 Mark III camera equipped with a Sony 50 mm F1.8 Lens and a 10 mm macro photography tube adapter. Additionally, microscopic images of selected seedlings were taken using a Leica M165 FC microscope and the Leica Application Suite (LAS) version 3.8.0. We measured root- and root hair length using ImageJ version 1.54k [42].

For root hair measurements, we selected seedlings with a primary root length in the range of 30 mm to 55 mm at the start of the treatment period to ensure comparable groups of 30 biological replicates each. We first measured the distance from the root origin to the start of the earliest visible root hair bulges (emerging root hairs forming small protrusions) individually for each unique seedling before the treatment period. However, at this position the epidermis cells have already established planar polarity and fully differentiated into root hair cells named trichoblasts [43]. To include this developmental cycle and ensure the entire measured root hair growth and development takes place during the treatment period, we added 1 mm to the root emergence to bulge distance on each root to define the point immediately before root hair emergence. After the treatment period, we measured the root hair length of 10 root hairs at the defined position on each of the roots and calculated their arithmetic mean.

**Fig. 1:**
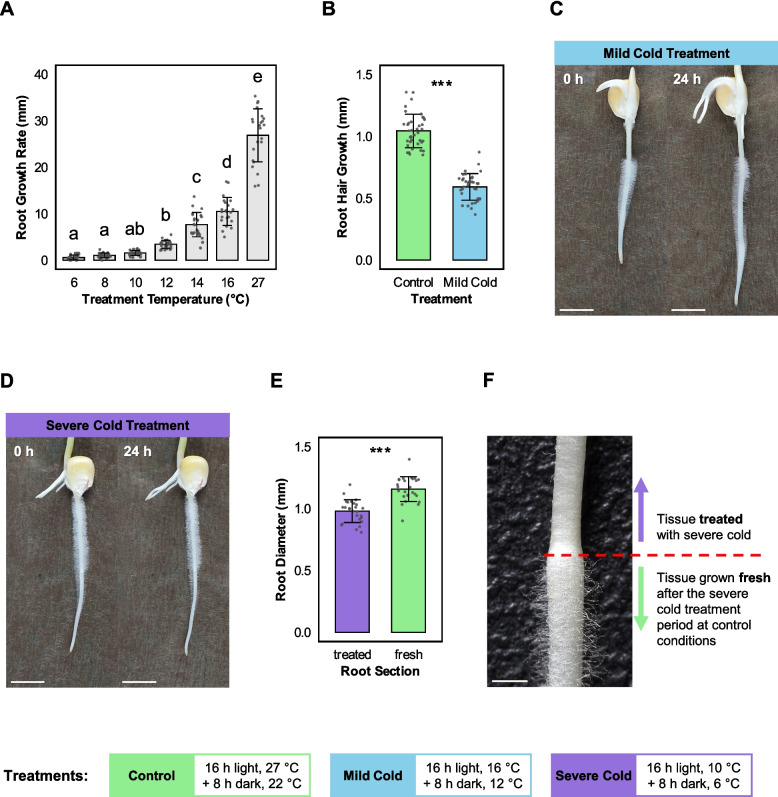
Temperature treatments affect primary root and root hair growth revealing severity dependent phenotypes. **A** Comparison of primary root growth over 24 h at different temperature regimes (6, 8, 10, 12, 14, 16, and 27 °C). Bars represent the mean ± standard error (SE), with 30 biological replicates per group. Statistical significance was determined by a one-way ANOVA followed by Tukey’s HSD post-hoc test (*p* < 0.05). Bars sharing the same letter are not significantly different. **B** Comparison of root hair growth during a 24 h treatment period of control treatment (16 h Light, 27 °C / 8 h dark, 22 °C) and mild cold (16 h Light, 16 °C /8 h dark, 12 °C) treatment. Error bars represent the mean ± standard error (SE) from 30 biological replicates per group. Statistical significance was determined by a paired t-test (***p < 0.001). **C** The pictures show the same exemplary root before (left side) and after (right side) 24 h of mild cold treatment. **D** The pictures show the same exemplary root before (left side) and after (right side) 24 h of severe cold treatment (16 h Light, 10 °C /8 h dark, 6 °C). **E** Comparing measurements of root diameter at thinnest part of the cold treated root immediately before the transition and the thickest part after the transition to the freshly formed root tissue that grew after the severe cold treatment. Error bars represent the mean ± standard error (SE) from 30 biological replicates per group. Statistical significance was determined by a paired t-test (***p < 0.001). **F** Image of a maize root after 24 h of severe cold stress, followed by 24 h under control conditions. The red dotted line marks the position of the root meristem at the end of the cold treatment period, which is now located in the mid-root region due to subsequent root growth under control conditions

For the root diameter measurements, we subjected 30 3-day-old seedlings first to 24 h of severe cold treatment (10 °C/6 °C, 16 h light/8 h dark), and subsequently to 24 h of control treatment (27 °C/22 °C, 16 h light/8 h dark). At the former position of the root tip (measured as root length before the start of the control treatment) a distinguishable transition between the cold treated tissue and the freshly grown tissue under control treatment formed based on the occurrence of root hair (Fig. 1F). We measured the diameter of each root 1 mm proximal and 1 mm distal of this position.

### Root hair harvest and sample preparation for RNA analysis

For transcriptome analyses, we germinated a set of seedlings under the same initial conditions (3 days at 27 °C/22 °C, 16 h light/8 h dark) and then subjected them to either a 1-day mild cold treatment (16 °C/12 °C; 16 h light/8 h dark), a 1-day severe cold treatment (10 °C/6 °C, 16 h light/8 h dark), or maintained them at control treatment for the same duration. Immediately after the treatment period, we harvested root hairs to capture their gene expression profiles. For each RNA sample, approximately 120-150 seedlings constituted a pooled biological replicate. We selected seedlings with primary root lengths between 2 cm and 6 cm, briefly dipped them into liquid nitrogen, and dislodged the root hairs with a pre-chilled spatula into a beaker of liquid nitrogen. The collected root hairs were later transferred into 1.5 mL microcentrifuge tubes and stored at −86 °C until RNA isolation.

### RNA isolation and sequencing

We prepared 15 RNA samples, representing 5 biological replicates for each of the three conditions (control, mild cold, severe cold). Total RNA was extracted using the RNeasy Plant Mini Kit (Qiagen, Hilden, Germany) with on-column DNase treatment following the manufacturer’s instructions. RNA quality and quantity were assessed with a NanoDrop spectrophotometer (Thermo Fisher Scientific, Waltham, MA, USA) and an Agilent 2100 Bioanalyzer (Agilent Technologies, Santa Clara, CA, USA). All samples had RNA Integrity Number (RIN) values ≥8 [44]. Libraries were constructed and sequenced by Novogene (Cambridge, UK) on a NovaSeq X Plus platform using a paired-end 150 bp strategy, yielding approximately 12 G raw data per sample.

### Data processing and alignment

Initial raw reads were trimmed and filtered by Novogene to remove adapter sequences and low-quality reads. We imported the resulting high-quality FASTQ files into CLC Genomics Workbench 24.0.1 (QIAGEN Bioinformatics, Aarhus, Denmark) and aligned them to the Ensembl Plants reference genome Zm-B73-REFERENCE-NAM-5.0 using the default parameters: mismatch cost = 2, insertion cost = 3, deletion cost = 3, length fraction = 0.8, similarity fraction 0.8, maximum number of hits for a read = 10 (Tab S2). The processed sequencing data have been deposited in the NCBI Sequence Read Archive (SRA) under BioProject accession number PRJNA1243569.

### Differential expression analysis

We imported raw read counts (Fig. S2) obtained from CLC Genomics Workbench into R Studio version 2024.4.2 running R version 4.4.1 [45] for differential expression analysis. Genes with fewer than 0.5 counts per million (CPM) in at least one sample were filtered out to reduce noise. Normalization and differential expression (DE) analysis were performed using the DESeq2 package [46], which estimates size factors for library normalization and models count data with a negative binomial distribution. Unless otherwise specified, default settings of DESeq2 were applied, including the Benjamini-Hochberg method for multiple testing correction. Pairwise contrasts were computed between mild cold versus control and severe cold versus control conditions (Tab. S3). Genes were considered significantly differentially expressed if they displayed an absolute log_2_ fold change (|log_2_ FC| ≥ 1) and an adjusted *p*-value (FDR) < 0.05. We visualized the sample relationships using principal component analysis (PCA) and generated plots using ggplot2 [47] and ComplexHeatmap [48].

### Gene Ontology enrichment, KEGG pathway enrichment and transcription factor family analysis

Gene Ontology (GO) term annotations for maize were obtained from MaizeGDB [49]. We conducted functional enrichment analyses for GO terms (Biological Process, Cellular Component, and Molecular Function) (Tab. S1), and Kyoto Encyclopedia of Genes and Genomes (KEGG) pathways (Tab. S4) using the clusterProfiler R package [50]. For each comparison (mild cold vs. control and severe cold vs. control), upregulated and downregulated DEGs were analyzed separately to identify overrepresented GO terms and KEGG pathways. Enrichment was assessed using a hypergeometric test, and *p*-values were adjusted for multiple testing using the Benjamini-Hochberg procedure to control the false discovery rate. Terms and pathways with an adjusted *p*-value < 0.05 were considered significantly enriched. We summarized and visualized GO terms using enrichplot and ggplot2, while KEGG pathway results were further examined and depicted using Pathview [51] to integrate differential expression analysis results onto pathway diagrams. As previously described [16], transcription factors (Tab. S5) were acquired from the Grass Transcription Factor Database (GrassTFDB) and their gene identifiers were converted to version 5 of the maize reference genome with the online tool Gene Center in MaizeGDB [49].

## Results

### Cold induced phenotypic alterations in maize roots reflect stress severity

To investigate how primary root growth of the inbred line B73 responds to different cold treatments compared to near optimal conditions for root growth, we germinated seedlings for three days under control conditions (27 °C/22 °C; 16 h light/8 h dark) and then subjected them for 24 h to different cold treatments (6 °C to 16 °C) or near optimal (27 °C) conditions (each 16 h light/8 h dark). We determined growth rates as the difference in primary root length before and after the 24 h treatment. All cold treatment conditions severely inhibited primary root growth compared to the control (Fig. 1A). At treatment temperatures of 10 °C and below primary root growth almost ceased with no significant differences between the treatments (Fig. 1A). At temperatures of 12 °C and above primary root growth increased progressively with raising temperature treatments, but was significantly inhibited compared to the control group grown at 27 °C (Fig. 1A).

Based on these findings, we focused on two distinct cold treatments that we defined as mild cold (16 °C/12 °C; 16 h light/8 h dark) and severe cold treatment (10 °C/6 °C; 16 h light/8 h dark) compared to the control treatment (27 °C/22 °C; 16 h light/8 h dark).

To assess the effects of mild cold stress on root hair elongation we subjected two groups of seedlings with comparable root length to each 24 h of mild cold and control treatment (see Methods). 24 h of mild cold treatment significantly reduced root hair elongation to 57% of that of the control (Fig. 1B). Apart from reduced elongation, development of the root was normal after 24 h of mild cold treatment (Fig. 1C).

Seedlings exposed to the severe cold temperatures showed almost no growth and development after 24 h of cold treatment (Fig 1A; D). We therefore subjected seedlings that were subjected to severe cold treatment (10 °C/6 °C; 16 h light/8 h dark) to a consecutive control treatment (27 °C/22 °C; 16 h light/8 h dark). We observed significant differences between the previously existing tissue subjected to severe cold and the tissue that newly developed after the end of the cold treatment period. The root diameter of the section of the root that developed immediately after the end of the cold treatment exceeded that of the preceding cold-treated section by 18% (Fig. 1E). Additionally, for all roots (n = 30), the section subjected to severe cold did not recover the ability for root hair elongation (Fig. 1F). The newly grown tissue that developed after the end of the severe cold treatment showed normal root hair development (Fig. 1F).

Overall, our experiments demonstrated that maize seedlings exhibit distinct morphological responses to mild and severe cold stress. While both types of cold stress inhibit primary root and root hair growth, the effects of mild cold treatments are limited and temporary while severe cold stress leads to a permanent arrest of cell extension in the affected tissue.

### Severity-dependent transcriptomic responses of maize root hair cells to cold stress

To investigate the transcriptomic responses underlying the phenotypic alterations to cold stress observed in maize roots, we subjected 3-day-old seedlings to control (27 °C/22 °C; 16 h light/8 h dark), mild cold (16 °C/12 °C; 16 h light/8 h dark) and severe cold (10 °C/6 °C; 16 h light/8 h dark) treatments. For RNA-sequencing we extracted RNA from root hairs sampled from shock frozen primary roots in five biological replicates, each pooled from 120 seedlings.

**Fig. 2:**
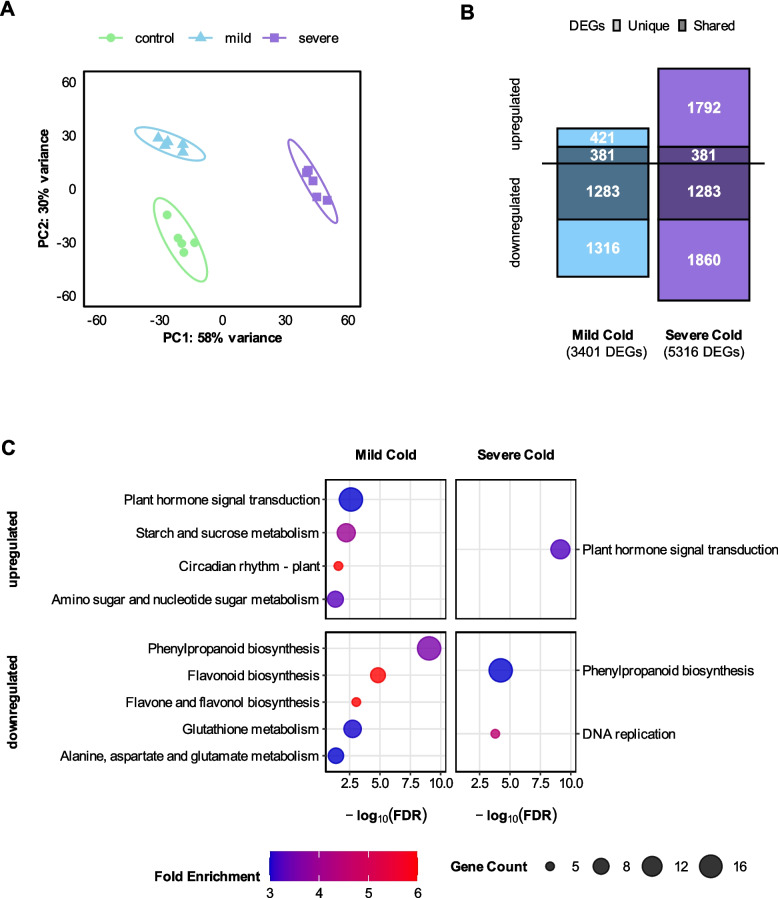
Severity-dependent transcriptomic responses in maize root hairs under cold stress. **A** Principal component analysis (PCA) of transcriptomes from root hair samples subjected to control (27 °C/22 °C; 16 h light/8 h dark), mild cold (16 °C/12 °C; 16 h light/8 h dark), and severe cold (10 °C/6 °C; 16 h light/8 h dark) conditions. The first two principal components (PC1 and PC2) explain 88% of the variance, with samples clustering closely by treatment. **B** Stacked bar plot illustrating the overlap of upregulated and downregulated differentially expressed genes (DEGs) after mild and severe cold treatments relative to control. DEGs were identified using an adjusted *p*-value < 0.05 and an absolute log_2_ fold change >1. **C** KEGG pathway enrichment analysis of upregulated and downregulated DEGs after mild and severe cold versus control treatment. Circle size represents the number of genes associated with each term or pathway, and color denotes fold enrichment. The x-axis represents the adjusted *p*-value (FDR), transformed as -log_10_(FDR), with larger values indicating higher significance. Terms and pathways were considered significantly enriched with an adjusted *p*-value < 0.05 and a log_2_ fold change >1

We conducted a principal component analysis (PCA) to assess the overall expression variance among the samples and to confirm that differences in gene expression were primarily due to the different treatments. The PCA plot (Fig. 2A) illustrates that the first two principal components account for 88% of the total variance. Samples clustered closely within their respective treatment groups, indicating high reproducibility and minimal technical variation.

By computing pairwise contrasts, we identified significant changes in gene expression (FDR <0.05 and |log_2_FC| ≥1) in response to mild and severe cold treatments compared to the control. The mild cold treatment yielded a total of 3,401 differentially expressed genes (DEGs) compared to the control, with a larger number of genes being downregulated (2,599) and a smaller number of genes being upregulated (802). Severe cold treatment yielded a larger total number of DEGs (5,316), again with more downregulated (3,143) than upregulated genes (2,173) (Fig. 2B).

Analyzing the overlap of DEGs between the mild and severe cold treatments revealed that both conditions shared some gene expression changes while others were unique. For upregulated genes, approximately 48% (381 of 802) of the genes upregulated under mild cold conditions were also upregulated under severe cold stress (Fig. 2B). For downregulated genes, approximately 49% (1,283 of 2,599) of the genes downregulated under mild cold conditions were also downregulated under severe cold stress (Fig. 2B).

The high degree of overlap between the differentially expressed genes from mild to severe cold stress points to conserved regulatory pathways. The substantially larger number of regulated genes under severe conditions indicates the further regulation of distinct, severe cold stress specific responses.

### Functional enrichment reveals biological processes and pathways associated with cold stress

To gain insights into the biological processes and pathways affected by cold stress in maize root hair cells, we performed KEGG pathway analyses, which mapped the differentially expressed genes (DEGs) identified under mild and severe cold treatments onto known metabolic and signaling pathways. In addition, we conducted Gene Ontology (GO) term enrichment analyses to identify overrepresented biological functions associated with these DEGs (see Methods). We also examined which transcription factor families were overrepresented among the differentially expressed genes after mild and severe cold treatment. 

**Fig. 3:**
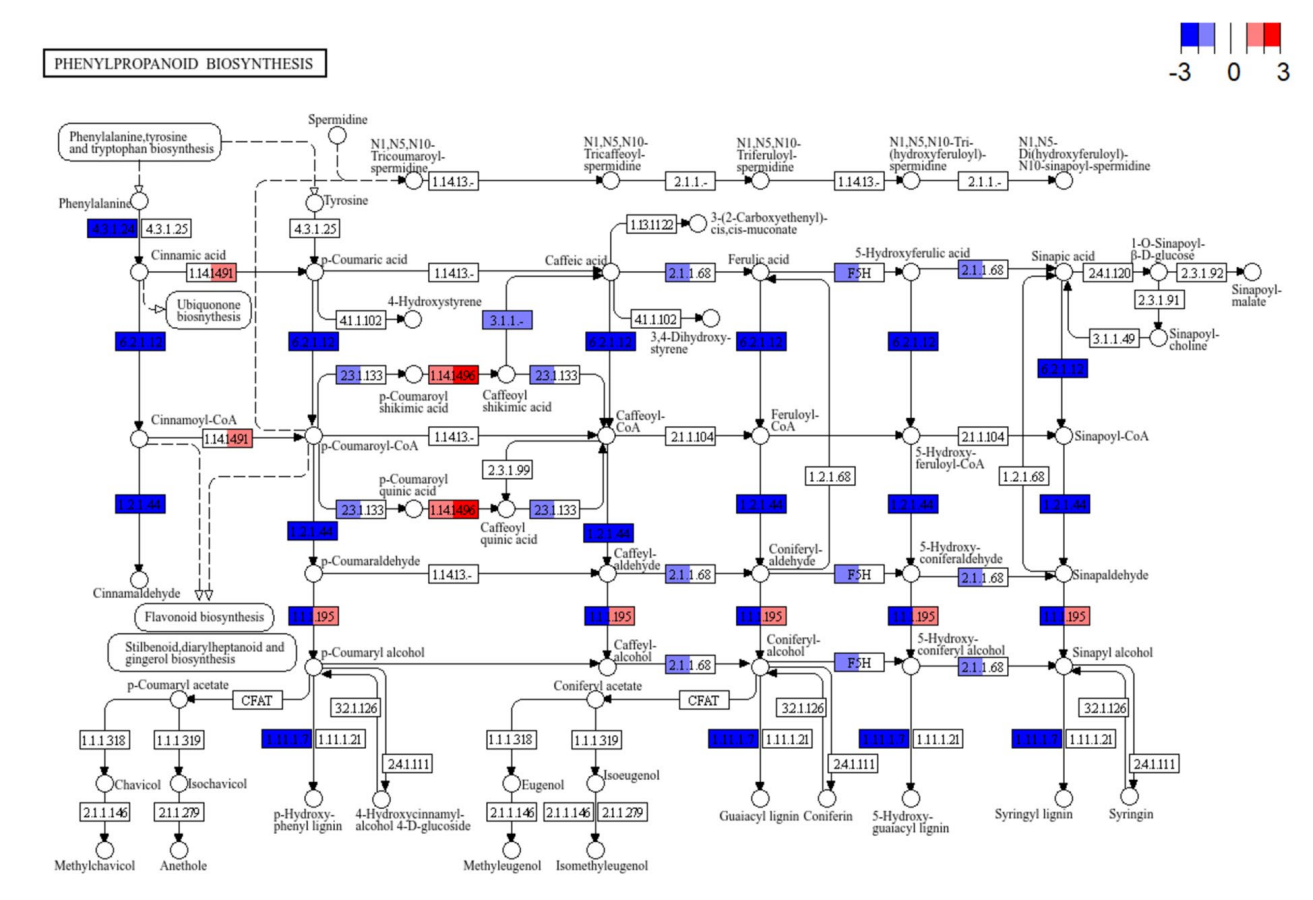
Transcript abundance changes in the KEGG “Phenylpropanoid Biosynthesis” pathway. Pathview visualization of differentially expressed genes under mild cold (left side of each tile) and severe cold (right side of each tile) treatment compared to control treatment. Genes are mapped onto the KEGG pathway zma00940 (Phenylpropanoid Biosynthesis). Color codes indicate the direction and magnitude of expression changes (log_2_ fold change; red for upregulation, blue for downregulation)

KEGG (Kyoto Encyclopedia of Genes and Genomes) pathway enrichment analysis revealed several significant pathways affected by cold stress in maize root hair cells (Fig. 2C). The phenylpropanoid biosynthesis KEGG pathway was significantly downregulated in both mild and severe cold treatments, with a more pronounced effect under mild cold stress, suggesting a reduction in secondary metabolite production. The phenylpropanoid metabolism plays an essential role in lignin biosynthesis and cell wall formation in plants. The Pathview visualization for the mild cold and severe cold DEGs (Fig. 3) illustrates widespread downregulation of genes involved in lignin and flavonoid biosynthesis, such as PAL, C4H, and 4CL enzymes. Furthermore, downregulated DEGs in the mild cold versus control comparison were enriched in GO terms related to secondary metabolite biosynthesis and defense responses, such as "lignin biosynthetic process" and "defense response to other organism". These GO terms are associated with the phenylpropanoid pathway and biotic stress responses (Fig. S1, Tab. S1).

**Fig. 4:**
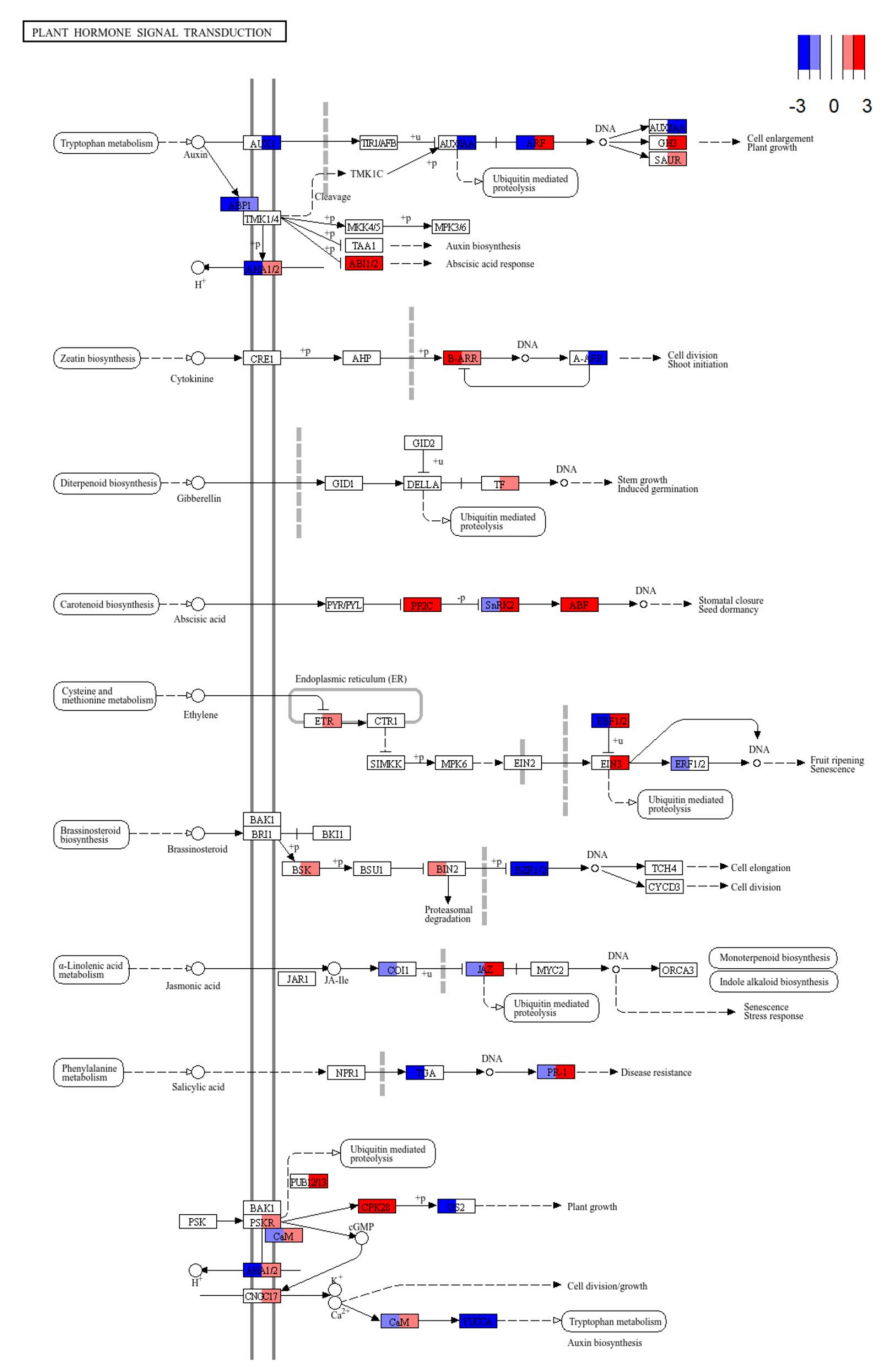
Transcript abundance changes in the KEGG “Plant Hormone Signal Transduction” pathway. Pathview visualization of differentially expressed genes under mild cold (left side of each tile) and severe cold (right side of each tile) treatment compared to control treatment. Genes are mapped onto the KEGG pathway zma04075 (Plant Hormone Signal Transduction). Color codes indicate the direction and magnitude of expression changes (log_2_ fold change; red for upregulation, blue for downregulation)

The Pathview visualization for plant hormone signal transduction showed differential regulation between mild and severe cold treatment (Fig. 4) compared to control conditions. Notably, certain hormone signaling pathways were regulated in opposite directions between the two conditions. Under mild cold stress, genes involved in cytokinin signaling were upregulated while these genes were downregulated under severe cold stress (Fig. 4). In contrast, genes associated with jasmonic acid, ethylene and salicylic acid signaling were downregulated under mild and upregulated under severe cold stress (Fig. 4). Hormonal signaling pathways were also significantly enriched in GO terms among the upregulated genes in both treatments but differed in the specific hormones involved, including auxin (GO:0009734), ethylene (GO:0009873) and abscisic acid (GO:0009738) related pathways (Fig. S1, Tab. S1).

Notably, both the mild cold versus control and severe cold versus control comparisons showed significant enrichment of upregulated DEGs in rhythmic processes, such as "circadian rhythm," suggesting adjustments in the internal clock under cold stress (Fig. S1, Tab. S1). Differences emerged in the stress response terms enriched in each dataset. Only in the severe cold versus control comparison, upregulated DEGs exhibited significant enrichment in abiotic stress responses, including "response to cold", "response to salt stress" and "response to hydrogen peroxide" forming a group of abiotic stress-related GO terms (Fig. S1, Fig. S1).

**Fig. 5:**
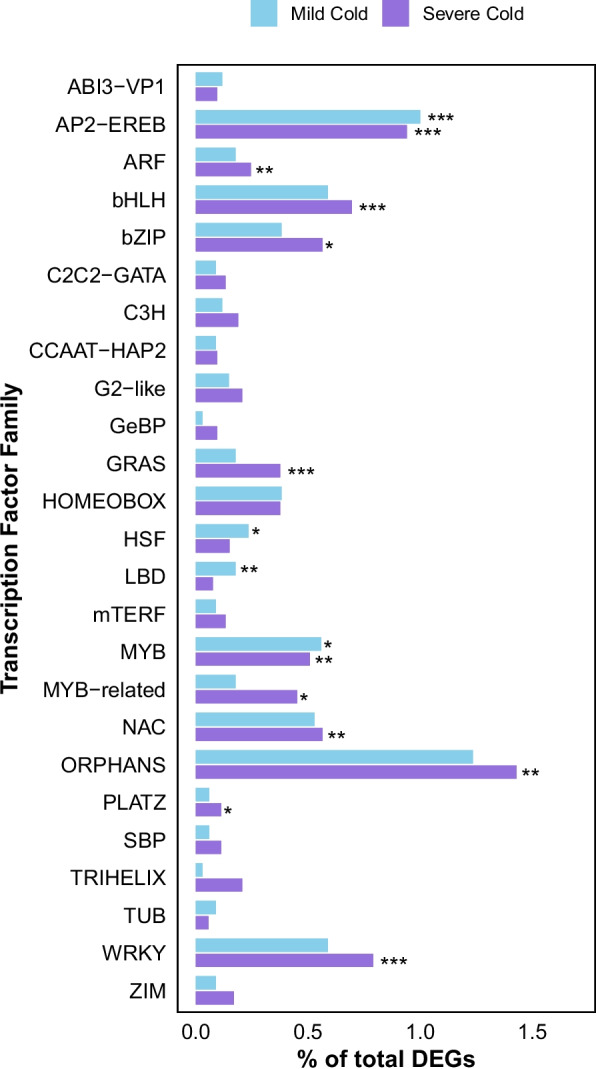
Differential expression of transcription factor families: Prevalence of differentially expressed genes (DEGs) after mild cold treatment (blue) and severe cold treatment (purple) in transcription factor (TF) families. Only families with ≥ 20 expressed members are shown. Bars represent the distributions of differentially expressed TFs as a percentage of all differentially expressed genes after each treatment (total DEGs mild cold = 3,401, total DEGs severe cold = 5,316). Significant deviations from the background distribution of DEGs for each treatment were calculated by Fisher's exact test (*p *≤ 0.05) and are indicated by asterisks

Environmental inputs, such as cold, modulate the activity and abundance of transcription factors which in turn regulate downstream gene expression. After the mild cold treatment, genes encoding AP2/EREB, Heat Shock Transcription Factors (HSTFs), Lateral Organ Boundaries Domain (LBD) and Myeloblastosis (MYB) transcription factors were significantly overrepresented among mild cold stress-responsive genes compared with all expressed genes (Fig. 5). After the severe cold treatment, in addition to the overall higher amount of total DEGs compared to the mild cold treatment, a significantly larger number of transcription factor families showed significant overrepresentation of severe cold stress-responsive genes compared with all expressed genes (Fig. 5).

Together, these findings suggest that the severity of the cold stress exerts distinct influences on cell wall related genes, hormone signaling, and transcriptional cold response regulation.

### Differential expression of genes modulating cell wall structure and root hair development under cold stress

**Fig. 6:**
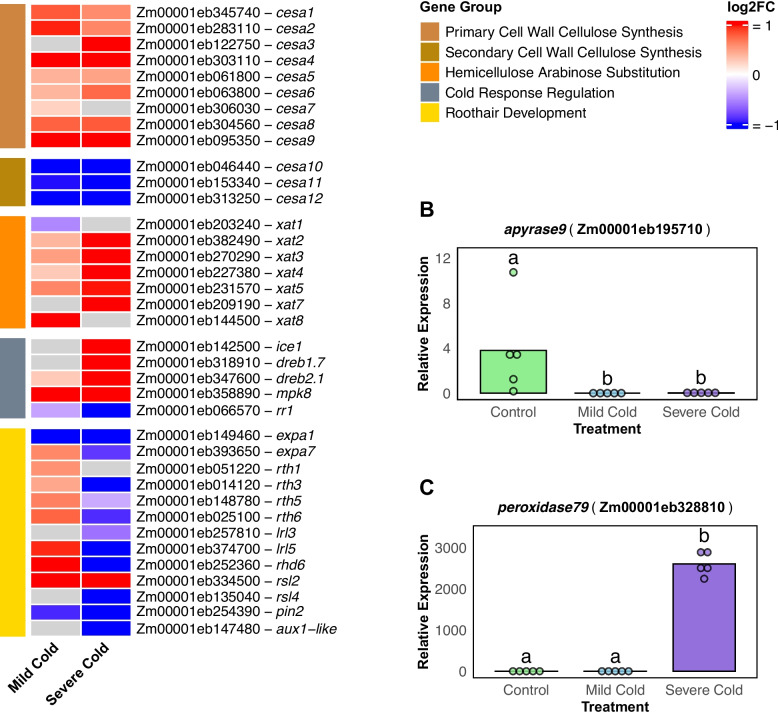
Expression patterns of genes involved in cell wall modification, root hair development, and cold-response pathways under cold stress. **A** Heatmap depicting log_2_ fold changes (as color coded tiles) in gene expression for selected significantly differentially expressed genes associated with cell wall structure, root hair formation, and cold stress regulation after mild- and severe-cold treatments relative to control treatment. Genes are grouped by functional category, indicated by colored bars at the left of the heatmap and in the legend. Gray tiles represent genes with differential expression that did not meet the adjusted *p*-value threshold (< 0.05). **B** Average counts per million (CPM) of *apyrase9* (Zm00001eb195710) after control, mild cold, and severe cold treatment, showing unique high expression under control and negligible expression under both cold treatments. **C** Average CPM of *peroxidase79* (Zm00001eb328810) under the same three conditions, indicating low expression after control and mild cold, but very strong induction after severe cold treatment

In the previous chapter, we identified significant regulation of cell wall related pathways, notably the downregulation of lignin precursors. To further explore cell wall modulation under cold stress, we examined the expression of key enzymes affecting cell wall composition (Fig. 6A).

Among the expansin gene family, *expansin1* and *expansin7* were the only genes with substantial expression across all three treatments. Under severe cold stress, both expansins were significantly downregulated, suggesting inhibited cell wall loosening essential for root hair tip growth (Fig. 6A).

Cellulose synthases (CESA) are crucial for cellulose synthesis in the cell wall. We observed a substantial upregulation of genes involved in primary cell wall cellulose synthesis (*cesa1 to 9*) under both cold treatments. Interestingly, genes associated with secondary cell wall cellulose synthesis (*cesa10 to12*) were significantly downregulated, indicating differential regulation of cellulose synthesis pathways between primary and secondary cell walls under cold stress. In contrast to the downregulation of cellulose synthases, β−1,4-xylosyltransferases, enzymes essential for xylan biosynthesis in the secondary cell wall, showed no significant change in regulation under mild cold stress and a slight upregulation under severe cold stress. Furthermore, under severe cold treatment, xylan α−1,3-arabinofuranosyl-transferases, which catalyze the addition of arabinofuranose residues to the xylan backbone and influence interactions with other cell wall components, were significantly upregulated (Fig. 6A). These findings suggest modifications in hemicellulose composition under severe cold stress.

We observed significant regulation of genes encoding known cold response transcription factors primarily under severe cold stress (Fig. 6A). Notably, *ice1* and *dreb1*, key regulators of cold-responsive genes, were upregulated only under severe cold treatment, indicating activation of cold-responsive pathways specifically in response to severe cold stress.

Genes essential for root hair development, such as *rth6* and *rhd6*, displayed contrasting expression patterns between mild and severe cold treatments (Fig. 6A). Under mild cold stress, these genes were significantly upregulated, suggesting promotion of root hair development. In contrast, they were significantly downregulated under severe cold stress, indicating inhibition of root hair growth under more extreme conditions.

The only apyrase gene detected in our dataset, *apyrase9*, was highly expressed in the control but was completely downregulated under both mild and severe cold treatments (Fig. 6B), indicating strong suppression in response to cold stress. Conversely, *prx79*, a peroxidase associated with abiotic stress responses, showed minimal expression under control and mild cold conditions but was among the most highly expressed genes under severe cold stress (Fig. 6C). This substantial increase suggests a pivotal role for *prx79* in the response to severe cold stress in maize root hairs.

Overall, these results demonstrate that mild and severe cold stresses drive distinct yet interconnected molecular adjustments in maize roots, influencing genomics pathways involved in cell wall restructuring, hormonal regulation, and root hair growth. These molecular patterns, together with the observed phenotypes, underscore the nuanced capacity of roots to modulate their development and physiology in accordance with the severity of the cold stress experienced.

## Discussion

Maize grown in temperate regions can encounter low early-season temperatures that impair plant vigor and overall seedling establishment [8]. In this study, we examined how varying intensities of cold stress induce distinct morphological and transcriptomic changes in maize root hairs. By subjecting seedlings of the inbred line B73 to two different cold treatments, we aimed to capture both shared and unique genomic responses that underpin root hair growth dynamics under cold stress. Our findings provide insights into which stress-responsive pathways become active under moderately low temperatures and which require more extreme conditions to be triggered.

### Morphological responses to different severities of cold stress

While the impact of mild and severe cold exposure on the morphology and transcriptome of young maize seedlings has been studied individually [19], our study comparatively dissected both, the differences in morphology and transcriptome between varying cold temperature treatments.

When transferred to mild cold conditions, young maize seedlings show significantly reduced primary root growth [52] and root hair elongation [16]. We demonstrated that these morphological changes associated with mild cold stress occur in the maize inbred line B73 down to temperatures of 12 °C. At a temperature of 5 °C, cell growth and cell division in the maize root apex nearly cease and cells in the former elongation zone behind the meristem mature and fail to continue growing when the roots are returned to warmer conditions [53]. We demonstrated that this near cessation of root growth already occurs at 10 °C for the inbred line B73 with no significant differences at lower temperatures. Returning the seedlings to warmer conditions after our chosen severe cold treatment led to morphological changes associated with severe cold stress as previously described [37, 53]. Furthermore, we determined that the permanent arrest of growth and development of root hairs corresponds with the root section treated with severe cold. Root hair growth in plants is primarily driven by cell elongation through tip growth, a process of targeted deposition of cell wall and membrane material at the growing tip [54]. Our results showed that the fate of root hairs can be used as an indicator for the severity of the cold stress experienced by the root, whereas temporary inhibition of root hair growth corresponds to mild cold stress and permanent arrest of root hair growth indicates severe cold stress.

### Transcriptome wide patterns reveal cold stress severity dependent gene regulation

Previous research revealed that the transcriptomic variance among different maize genotypes [15] or cell types [16] can overshadow the differences induced by cold versus control treatments. Moreover, the epidermis displays the strongest transcriptomic response to cold [16], highlighting root hairs as an ideal model for investigating cold induced gene expression. To avoid masking transcriptomic signals, we focused on this single cell type in the maize inbred line B73. In contrast to those earlier findings, where principal component analysis (PCA) underscored genotype or cell type distinctions [15, 16], our PCA (Fig. 2A) shows that samples cluster tightly by treatment. This outcome indicates that the observed gene expression variance arises predominantly from the distinct cold treatments rather than any confounding factors, thereby affirming the robustness of our data.

A broad survey of maize cold response studies [19] showed that gene expression patterns vary markedly depending on the exact temperature regime used. Consistent with our results, maize response to moderate cold involves general downregulation of gene expression [19]. In contrast, the response to severe cold manifests as induction of expression of genes related to major signaling pathways and to the transcription machinery [19]. The results from our DEG analysis indicate that mild and severe cold conditions trigger both shared and unique sets of differentially expressed genes in maize root hairs (Fig. 2B). Interestingly, the overlap of DEGs between mild and severe cold (49%) is significantly smaller compared to other abiotic stresses such as drought, where 90% of DEGs responding to mild water deficit also responded to severe water deficit [55]. Together, these observations suggest that once cold stress surpasses a certain threshold, maize root hairs mount a broader network of adaptive mechanisms, consistent with the sharper phenotypic changes observed under severe cold treatment.

### Cell wall remodeling and oxidative stress

Lower temperatures inhibit maize root growth by reducing cell wall extensibility rather than turgor pressure [37]. Under cold stress, we noted a broad suppression of the phenylpropanoid pathway, including genes for enzymes such as PAL, C4H, and 4CL (Fig. 3), which are essential for lignin biosynthesis [56]. Cold stress reduces lignin content in poplar by regulating key genes and metabolic intermediates in lignin biosynthesis [57]. Lignin strengthens and waterproofs plant secondary cell walls [58]. Therefore, reduced lignin biosynthesis might help maintain a flexible and permeable cell wall. Notably, we also observed elevated xylan α−1,3-arabinofuranosyl-transferase expression (Fig. 6A). The addition of arabinofuranosyl side chains increases the hydrophilic nature of xylan, improving water retention and elasticity of the cell wall [59].

*Cesa8* is upregulated in maize root hairs experiencing mild cold stress [16]. Several members of the *cesa* gene family were upregulated under both mild cold and severe cold. Interestingly, *cesa10*, *11*, and *12* were significantly downregulated at both mild cold and sever cold treatment. *cesa10*-*12* are co-expressed in maize roots and are responsible for secondary cell wall biosynthesis. Mutants for *cesa10* to *12* exhibit a reduced cell wall thickness and cellulose content [60].

Expansins accumulate in the tip region of growing root hairs and facilitate cell wall modification [61]. *EXP7* is root hair specific and required for root hair elongation in Arabidopsis [62]. It is positively regulated by the transcription factor ROOT HAIR DEFECTIVE6 (RHD6) [63]. We observed that the expression changes of the maize homolog *exp7* [64], correlate with the changes in expression of *rhd6* under both mild cold and severe cold treatment (Fig. 6A). The associated downregulation of *rhd6* and *exp7* under severe cold treatment in conjunction with the absence of morphological root hair elongation indicates that *exp7* likely serves a similar function in maize as it does in Arabidopsis.

*Apyrase9* was the only apyrase expressed in our root hair dataset. A*pyrase8*, *apyrase9*, and *apyrase12* exhibit specific expression in roots, indicating a potential role for these genes in root-related processes [65]. We observed near-complete repression of *apyrase9* under both cold treatments (Fig. 6B). Apyrase suppression raises extracellular ATP (eATP) levels and induces gene expression and cell wall changes characteristic of stress responses [66]. Elevated eATP acts as a signaling molecule, interacting with reactive oxygen species, nitric oxide, and phytohormones to mediate stress responses [67]. While cold stress induced transcription inhibition of *apyrase5*, *apyrase8*, *apyrase11*, *apyrase13* and *apyrase15* has been previously reported [65], our findings indicate a possible root hair specific regulation response for *apyrase9.*

Chilling conditions frequently lead to excessive accumulation of reactive oxygen species (ROS), prompting plants to boost antioxidant defenses [68]. Under severe cold, *peroxidase79* emerged as one of the most strongly induced genes (Fig. 6C). Peroxidases of this family typically scavenge hydrogen peroxide and play roles in cell-wall strengthening, both of which can mitigate oxidative damage while also stabilizing newly formed cell-wall polymers [69, 70]. In contrast, mild cold treatment yielded a comparatively muted induction of this peroxidase, suggesting that moderate chilling may not drive ROS to critical damaging levels. Collectively, our findings underscore that maize root hairs undergo extensive cell wall remodeling in response to different severities of cold stress.

### Hormone signaling and transcription factor modulations

Our KEGG and Gene Ontology (GO) analyses revealed that plant hormone signal transduction pathways played a critical role in determining root hair fate under mild and severe cold treatment (Figs. 2C, 4). Plant hormones can regulate abiotic stress responses in plants through control of stress-responsive transcription factors (TFs) [71]. In turn, stress-responsive TFs can bind to the promoter of stress-responsive genes and regulate their expression [72, 73]. AP2/EREB transcription factors, particularly from the DREB subfamily, are major regulators of abiotic stress responses such as cold, drought, heat and salt stress [74]. In our expression analysis we found that this TF-family had a significantly higher amount of DEGs compared to the background of expressed genes after both the mild and severe cold treatment (Fig. 5), supporting the notion that members of the AP2/EREB family play an essential role in cold stress response [75, 76]. However, the patterns of the transcriptomic changes induced by mild and severe cold stress are fundamentally different [19]. Apart from the AP2/EREB TF-family, the severe cold treatment yielded a higher total amount of differentially expressed TFs and more TF-families with a significant overrepresentation of DEGs compared to the background of all expressed genes (Fig. 5). Thus, our observations confirm that severe cold activates a markedly broader transcriptional program than mild cold.

In Arabidopsis, the transcription factors RHD6 and RSL1 are active in early development and direct root hair formation by regulating the activity of the transcription factors RSL2, RSL3, RSL4, and LRL3 [77, 78]. We observed an upregulation of maize *rhd6* along several other root hair development and elongation related genes under mild cold treatment. By contrast, we observed a downregulation of *rhd6* (also referred to as *rsl1* [64]) along several other root hair development and elongation related genes under severe cold treatment (Fig. 6A). The shifts in these known regulatory pathways correlate with our morphological observation of still having significant root hair growth under mild cold and absolute inhibition of root hair growth under severe cold.

In Arabidopsis auxin triggers root hair development via *RSL2* and *RSL4* independently of *RHD6/RSL1* [77]. *RSL4* subsequently activates a set of genes expressed specifically in root hairs and/or required for root hair growth, such as *EXPANSIN A7* (*EXP7)* [77]*.* In Arabidopsis, the PIN-formed protein2 (PIN2) auxin efflux carrier is necessary for root hair growth [79]. We observed significant downregulation of the maize *pin2* (also referred to as *pin1b* [80]) and *aux1* (*auxin transporter-like1*; also referred to as *atl1* [81]) genes under mild cold and even stronger downregulation under severe cold (Fig. 6A). Taken together, the pronounced downregulation of these auxin transporters under cold likely reflects a broader disruption of auxin signaling, thereby inhibiting normal root hair development.

Jasmonate signaling also influences root hair development, with Jasmonate ZIM-domain proteins (JAZ) physically interacting with and inhibiting *RHD6* in Arabidopsis [82]. In our study, JAZ was significantly upregulated under severe cold and downregulated under mild cold [Fig. 4] corresponding to the observed expression changes of *rhd6*. Our findings indicate that JAZ expression in maize likely serves a similar function regarding the inhibition of *rhd6*.

The ICE-CBF/DREB1-COR transcriptional pathway plays a critical role in modulating cold stress responses in Arabidopsis [21, 83]. We observed an upregulation of *ice1* [84] and *dreb1.7* [85] specifically under severe cold conditions (Fig. 6A).

In Arabidopsis, cytokinin signaling promotes root hair elongation via the B-type response regulator *ARABIDOPSIS RESPONSE REGULATOR 1* (*ARR1*) by directly upregulating the transcription factor *ROOT HAIR DEFECTIVE 6-LIKE 4* (*RSL4*) [86]. In maize root hairs a homolog of the *rsl4* gene was previously identified [64]. Moreover, in maize seedlings *response regulator1* (*rr1*) positively regulates the expression of *dreb1* and cellulose synthase genes (*cesa*) to enhance cold tolerance [87]. Consistent with our morphological observations *rr1* was significantly upregulated under mild cold treatment and downregulated under severe cold treatment in our dataset. Moreover, *rsl4* was not differentially expressed under mild cold but was significantly downregulated under severe cold (Fig. 6A). In maize seedlings, the gene for *mitogen-activated protein kinase 8* (*mpk8)* is activated by cold stress and phosphorylates *rr1* which promotes the ubiquitin-mediated degradation of *rr1* [87]. We observed upregulated expression of *mpk8* under both cold treatments confirming an activation by cold.

Ethylene inhibits root elongation and promotes root hair formation in maize [88]. The transcription factor ETHYLENE-INSENSITIVE 3 (EIN3), a key component of the ethylene signaling pathway in maize [89], was upregulated under severe cold treatment (Fig. 4). Likewise, under mild cold treatment, we observed a significant inhibition of *ein3 binding f-box1/2* (*ebf1/2*) which targets *ein3* for degradation in the absence of ethylene (Fig. 4) [90]. In Arabidopsis EIN3 interacts with RHD6 to promote root hair elongation by coactivating *RSL4* [91]. Moreover, EIN3 negatively regulates the expression of CBFs and type-A Arabidopsis response regulators (ARRs) by binding to specific elements in their promoters [92]. Accordingly, we also observed a downregulation of maize type-A ARRs under severe cold treatment (Fig. 3).

Overall, our results highlighted concordant downregulation of know genes essential for root hair growth regulation in maize, such as *rhd6* [64], *lrl5* [93] and *roothairless 6* (*rth6*) [94] in the root hairs of seedlings subjected to severe cold, matching the observed morphological growth arrest. In contrast, under mild cold treatment the same root hair regulating genes are upregulated (Fig. 6A). However, the root hair growth rate under mild cold did not match that of the control treatment, indicating additional factors are limiting the potential for growth.

As previously reported [64], we also did not find *rsl3* expression in our samples and *rsl2* was only weakly expressed with expression correlating with neither observed root hair elongation nor known regulatory mechanisms. Therefore, our findings indicate that *rsl2* and *rsl3* likely do not serve the same regulatory function for root hair elongation in maize as they do in Arabidopsis.

## Conclusion

This study revealed how mild and severe cold trigger distinct transcriptomic programs in maize root hair cells. Mild cold permits limited elongation through targeted regulatory shifts, whereas severe cold activates broader stress pathways that arrest growth. Unlike most earlier transcriptome studies that examined just one chilling temperature, our comparison of two cold severities uncovers regulatory nodes whose activation clearly depends on stress intensity. These severity dependent shifts, especially in hormone signaling and cell wall remodeling, offer practical markers and potential breeding targets for cold tolerant maize. Because the data are root hair specific, future work should extend the analysis to other tissues and verify whether the severity linked signatures translate to whole plant performance under field conditions.

## Supplementary Information

Supplementary Material 1.

Supplementary Material 2.

Supplementary Material 3.

Supplementary Material 4.

Supplementary Material 5.

Supplementary Material 6.

Supplementary Material 7.

## Data Availability

The dataset generated and analyzed during the current study is available in the NCBI SRA repository under BioProject accession number PRJNA1243569 (https://www.ncbi.nlm.nih.gov/bioproject/PRJNA1243569). All data generated or analyzed during this study are included in this published article and its supplementary information files.

## Abbreviations

CBF: C-repeat binding factor
COR: Cold-regulated (genes/proteins)
CPM: Counts Per Million
DEG: Differentially Expressed Gene
FDR: False Discovery Rate
GO: Gene Ontology
KEGG: Kyoto Encyclopedia of Genes and Genomes
PCA: Principal Component Analysis
ROS: Reactive Oxygen Species
TF: Transcription Factor
