# Supplementary figures and images for: Differential cold stress intensities drive unique morphological and transcriptomic changes in Zea mays root hairs
Source: BMC Genomics. 2025 Sep 22;26:805. doi: 10.1186/s12864-025-12001-1 (PMC12452017; doi:10.1186/s12864-025-12001-1)

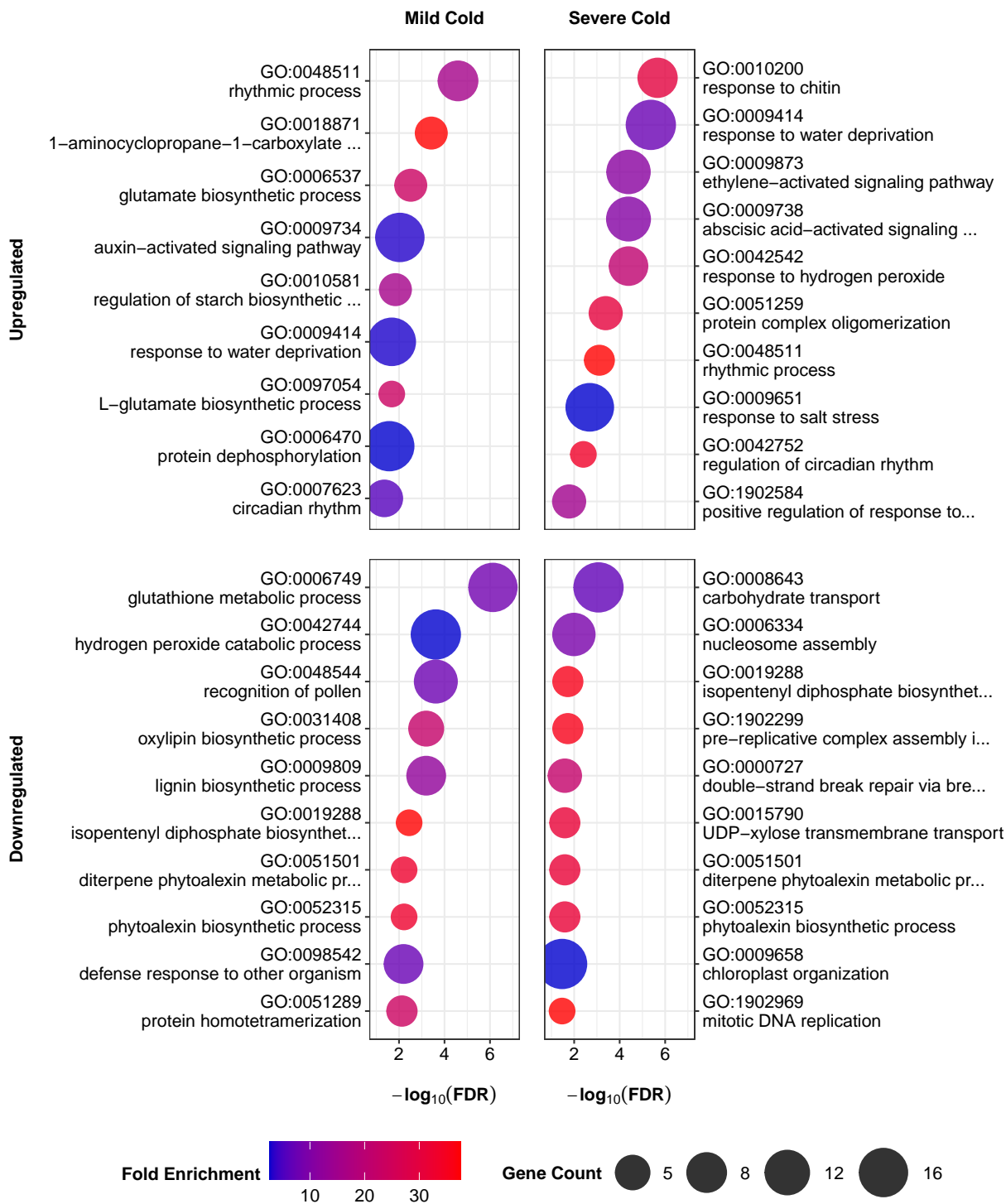

Supplement: Supplementary file 1 — Supplementary Material 1. [file 12864_2025_12001_MOESM1_ESM.pdf]

# Total Raw Read Counts

Control Treatment   Mild Cold Treatment   Severe Cold Treatment

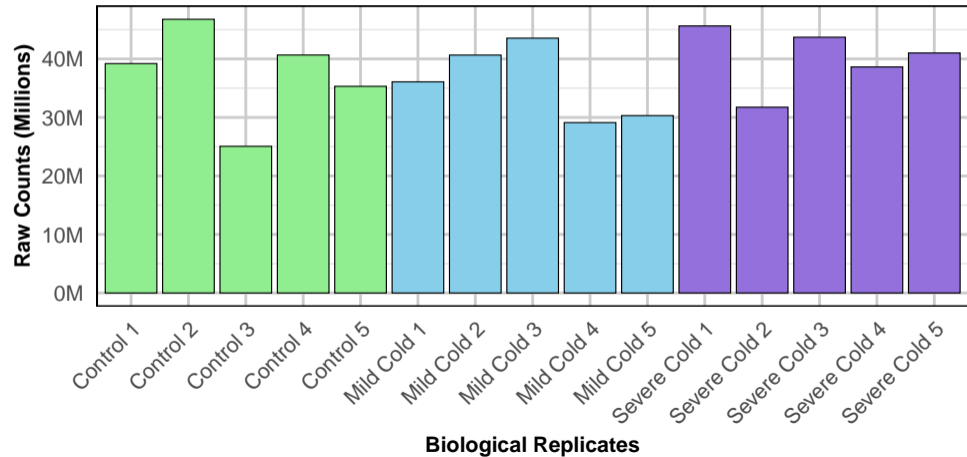

Supplement: Supplementary file 2 — Supplementary Material 2. [file 12864_2025_12001_MOESM2_ESM.pdf]
