# Supplementary material for: Differential cold stress intensities drive unique morphological and transcriptomic changes in Zea mays root hairs
Source: BMC Genomics. 2025 Sep 22;26:805. doi: 10.1186/s12864-025-12001-1 (PMC12452017; doi:10.1186/s12864-025-12001-1)
Supplement: Supplementary file 4 — Supplementary Material 4. [file 12864_2025_12001_MOESM4_ESM.docx]

**Table S2:** Overview of RNA-sequencing output aligned to the Ensembl Plants reference genome Zm-B73-REFERENCE-NAM-5.0.

| **Sample** | **Replicate** | **Total Fragments^1^** | **Uniquely Mapped Fragments^1^ (#)** | **Uniquely Mapped Fragments^1^ (%)** |
| --- | --- | --- | --- | --- |
| control | 1 | 61,063,163 | 43,348,714 | 70.99 |
|  | 2 | 62,742,481 | 50,840,578 | 81.03 |
|  | 3 | 40,982,616 | 27,574,302 | 67.28 |
|  | 4 | 62,164,562 | 44,839,284 | 72.13 |
|  | 5 | 49,787,708 | 39,126,306 | 78.59 |
| mild cold | 1 | 53,554,154 | 40,175,328 | 75.02 |
|  | 2 | 57,707,746 | 44,872,579 | 77.76 |
|  | 3 | 60,686,440 | 48,119,971 | 79.29 |
|  | 4 | 40,328,221 | 32,077,840 | 79.54 |
|  | 5 | 45,541,961 | 33,729,845 | 74.06 |
| severe cold | 1 | 57,861,250 | 50,804,403 | 87.80 |
|  | 2 | 42,345,679 | 35,807,739 | 84.56 |
|  | 3 | 55,446,425 | 48,786,105 | 87.99 |
|  | 4 | 51,872,035 | 43,446,035 | 83.76 |
|  | 5 | 54,435,572 | 46,383,993 | 85.21 |
| **Average** | | 53,101,334 | 41,995,535 | 79.00 |
| **Minimum** | | 40,328,221 | 27,574,302 | 67.28 |
| **Maximum** | | 62,742,481 | 50,840,578 | 87.99 |

^1^ For paired-end libraries, each “fragment” corresponds to a pair of reads derived from the same cDNA insert.
